# The Effect of a Masticatory Muscle Training Program on Chewing Efficiency and Bite Force in People with Dementia

**DOI:** 10.3390/ijerph19073778

**Published:** 2022-03-22

**Authors:** Julia Jockusch, Sebastian Hahnel, Bernhard B. A. J. Sobotta, Ina Nitschke

**Affiliations:** 1 Department of Prosthodontics and Materials Science, Gerodontology Section, University of Leipzig, 04103 Leipzig, Germany; sebastian.hahnel@medizin.uni-leipzig.de (S.H.); bernhard.sobotta@medizin.uni-leipzig.de (B.B.A.J.S.); ina.nitschke@medizin.uni-leipzig.de (I.N.); 2 University Research Priority Program “Dynamics of Healthy Aging”, University of Zurich, Andreasstrasse 15/Box 2, 8050 Zurich, Switzerland; 3 Clinic of General, Special Care and Geriatric Dentistry, Center of Dental Medicine, University of Zurich, 8032 Zurich, Switzerland

**Keywords:** dementia, chewing function, bite force, physiotherapy, mastication, mild cognitive impairment, chewing efficiency

## Abstract

Until now, no study has investigated the effects of masticatory muscle training on chewing function in people with dementia. This study aimed to investigate whether physiotherapeutic exercises for the masticatory muscles have an influence on chewing efficiency and bite force in people with dementia. In a clinical trial with stratified randomization subjects were assigned to three groups based on the Mini Mental State Examination (MMSE: group 1—28–30, group 2—25–27, group 3—18–24). Each group was divided into an experimental (ExpG, intervention) and control group (ConG, no intervention). As intervention a Masticatory Muscle Training (MaMuT) (part 1: three physiotherapeutic treatments and daily home exercises, part 2: daily home exercises only) was carried out. Chewing efficiency and bite force were recorded. The MaMuT influenced the masticatory performance regardless of the cognitive state. Bite force increased in ExpG 1 and 2. Without further training, however, the effect disappeared. Chewing efficiency increased in all ExpG. After completion of the training, the ExpG 2 and 3 showed a decrease to initial values. Subjects of ExpG 1 showed a training effect at the final examination, but a tendency toward the initial values was observed. ExpG 3 seemed to benefit most from the physiotherapeutic exercises in terms of improving chewing efficiency by the end of the intervention phase. ExpG 1 showed the greatest gain in bite force. The MaMuT program is a potential method of improving masticatory performance in people with cognitive impairment or dementia when used on a daily basis.

## 1. Introduction

The chewing function in humans is a complex neurophysiological mechanism [1,2,3,4,5]. Any kind of changes in the structures involved can alter the effectivity of the masticatory process [6]. Pathological changes in the masticatory musculature may be the cause of pain, of craniomandibular dysfunctions due to over-function, or contribute to malnutrition in the case of under-function.

Chewing function can be described by subjective chewing ability, objective chewing efficiency, and bite force. The chewing ability describes the subjective chewing ability of a person, which is determined through questionnaires [7]. Chewing efficiency refers to the objectively measurable chewing performance that can be reproducibly and objectively evaluated by tests [8,9,10,11,12,13,14,15,16,17,18]. Chewing efficiency can be negatively influenced by various factors such as a reduced number of teeth, occlusal surfaces [19,20,21,22,23,24,25], or antagonistic contacts [26]. The incorporation of dentures can only partially compensate for the reduction in chewing efficiency induced by tooth loss [27]. Furthermore, there is a correlation between the quality of dentures [11,28], their adaptation to changed oral situations (e.g., relinings) [29], and the type of denture [11,30,31,32,33] on chewing efficiency. A significant dependence of chewing efficiency on saliva quantity and consistency [34] but also on factors such as maximum bite force, the function of lip, cheek, tongue, and the soft palate [35,36] was shown. The bite force provides information about the physiologically possible force of the subject to comminute any chewables. It can be influenced by strength of the masticatory muscles, condition of the periodontal tissues, temporomandibular joints, and subjective perception [37,38]. Bite force is often directly related to masticatory performance [39,40].

Numerous studies demonstrated that poorer mastication is associated with lower cognitive function [41,42,43,44,45,46,47] and is a risk factor for having dementia or mild memory impairment, or for the incidence of dementia [48,49,50]. In addition, the physiology of aging leads to atrophy of the masticatory muscles (sarcopenia, hypoactivity of the chewing muscles) [51]. This process is further intensified by tooth loss [52] and results in a decrease of the maximum jaw closing force [8,53,54]. Furthermore, impaired motor skills in people with dementia appear to contribute to a reduced chewing efficiency [49] and an overall impaired masticatory function [49,55]. The negative effect which reduced cognitive abilities have on motor skills is more pronounced than the negative influence of a poor dentition on the cognitive state [56]. Reduced masticatory performance in combination with impaired motor skills [57,58] leads to a reduction of the cerebral blood flow and brain activity and thus promotes dementia [55]. It has been shown that mastication might have a preventive effect on cognitive function [59,60], in addition to other physical activities [61].

It is assumed that training of the masticatory muscles is comparable to training of any other muscle which induces a change in muscle fiber size and composition. This in turn will increase the strength of the muscle and the resistance to fatigue [62,63,64,65]. Several studies showed an increase in bite force [37,38,66,67,68,69,70,71,72] and saliva secretion [72,73] with the help of jaw training exercises. Thompson et al. (2001) stated that an increase in bite force can be achieved by isometric training (clenching against a soft maxillary splint, 1 min per day for 6 weeks). Nevertheless, they could not find any significant differences in the increase of the bite force between the test (+37% compared to baseline) and the control group (+25% compared to baseline) [38]. Most studies showing the effects of masticatory muscle training on chewing include only younger, healthy subjects [37,38,67,68,71]. Only some studies examined masticatory muscle training in older people [72,74]. As an increase in bite force seems to be easily developed by training, it is suggested that a training of the masticatory muscles, which involves both isotonic and isometric exercises, might be effective [71]. To the authors best knowledge, no study so far has investigated professional masticatory muscle training for improving bite force as well as chewing efficiency in people with dementia.

The aim of this study was to clarify whether physiotherapeutic exercises for strengthening the masticatory muscles have an influence on chewing efficiency and bite force. Furthermore, possible differences in the effectiveness of physiotherapeutic exercises for strengthening the chewing muscles depending on the cognitive state are to be investigated.

The authors hypothesized that (a) physiotherapeutic exercises for strengthening the masticatory muscles have an effect on the sense of improving bite force but not chewing efficiency (in all subjects) and (b) physiotherapeutic exercises are not as effective in people with onset of dementia and mild dementia compared to patients without cognitive impairment.

## 2. Materials and Methods

The available data in this analysis is part of the Oral Health, Bite Force and Dementia Study (OrBiD Study) (clinicaltrials.org number: NCT03775772) [75,76,77,78].

### 2.1. Study Population

Subjects aged 60 and over who had sufficient knowledge of German to be able to follow the instructions and examinations were included in the study, regardless of their cognitive abilities. Subjects with acute oral processes requiring emergency treatment were excluded until emergency treatment had been successfully completed. Subjects with signs and symptoms of cranio-mandibular dysfunction were excluded. Subjects had to have at least one antagonistic contact (including prosthetic restoration) per side of the jaw and no non-occlusion in the posterior region.

Additionally, subjects were excluded if they suffered from (a) physical limitations in the upper body due to musculoskeletal or neuromuscular conditions (e.g., arthritis, conditions after stroke with impairment of motor skills, and facial nerve paralysis, paralysis of the arms, etc.), or (b) congenital mental disabilities (e.g., Down syndrome, cerebral paresis).

### 2.2. Study Design

The study was a clinical trial with an intervention. Randomization was performed with stratification as dementia is expected to have a major impact. The subjects of the OrBiD study were assigned to one of five evaluation groups after the first examination based on the Mini Mental State Examination (MMSE) [79] (group 1—no dementia (noDem, MMSE 28–30); group 2—mild cognitive impairment (mCI, MMSE 25–27); group 3—mild dementia (mDem, MMSE 18–24); group 4 moderate dementia (MMSE 10–17) and group 5—severe dementia (MMSE ≤ 9)). Each evaluation group was divided into an experimental group (ExpG) (*n* = 12) and control group (ConG) (*n* = 12). The evaluation groups four and five were excluded from this part of the study due to severe cognitive impairment [75,76,77,78].

Further stratification of the study population with regard to other influencing factors (e.g., dental and denture status) was not attempted.

### 2.3. Data Collection

Subjects of the ExpG received an intervention and were evaluated at baseline (T0), 3 months (T1) and 6 months (T2) after starting the intervention, and 12 months after T0 (T3). Subjects of the ConG received no intervention and were evaluated at T0 and T3 only [75,76,77,78] (Supplementary Material I).

Socio-demographic data were recorded for all subjects.

Additionally, in a dental assessment, the number of teeth, number of support zones, and the tooth and denture status were recorded separately for the upper and lower jaw. The tooth and denture status are the result of the combination of the tooth status (categories: fully edentulous, partially edentulous, edentulous) and the denture status (categories: no denture available, removable denture, fixed denture). The tooth and denture status were recorded in five categories (1—fully dentate, no dentures or fixed dentures, 2—partially dentate, fixed dentures, 3—partially dentate, removable denture, 4—partially dentate, no dentures available, 5—edentulous, removable dentures).

The Mini Mental State Examination (MMSE, maximum score 30 points) [79], Barthel Index—activities of daily living (maximum score 100) [80], Mini nutritional assessment (MNA, maximum score 30 points, 17–23.5 points indicate being at risk of malnutrition, less than 17 points indicate a poor nutritional status) [81], and Body Mass Index (BMI) [kg/m^2^] were assessed.

The MMSE was collected at all evaluation time points. It was planned that subjects with a change in MMSE value during the study period that might have required a change in group assignment would be replaced by new subjects. However, no change in group assignments were necessary during the study.

The bite force (in Newton, N) was recorded at maximum voluntary clenching with the occlusal force meter GM 10^®^ (Morita, Nagano Keiki, Higashimagome, Ohta-ku, Tokyo, Japan) according to the procedure described in the literature [82]. The maximum occlusal force (MOF) of all measurements was included in the analysis. Chewing efficiency was determined by means of the color mixing ability test using Hue-check Gum^®^ (Orophys GmbH, Muri b. Bern, Switzerland) according to Schimmel et al. [15]. The subjective visual assessment (5-step ordinal subjective assessment scale (SAS): SAS1—chewing gum not mixed, impressions of cusps or folded once; SAS2—large parts of chewing gum unmixed; SAS3—bolus slightly mixed, but bits of unmixed original color; SAS4—bolus well mixed, but color not uniform; SAS5—bolus perfectly mixed with uniform color) and an opto-electronically analysis with the ViewGum^®^ software (www.dhal.com, accessed on 30 January 2022) was carried out. The program calculates the hue value as a ratio of the unmixed fraction of the chewing gum to the total pixel number in a fixed size template as variance of hue (VOH) which is a measure of the chewing efficiency. Inadequate mixing of colors as an expression of poor chewing efficiency will result in high VOH and vice versa [15,83].

All clinical procedures and evaluations were performed by a single investigator.

### 2.4. Intervention in the Experimental Group

The intervention was a Masticatory Muscle Training (MaMuT) which aims to strengthen the masticatory muscles and to improve coordination during chewing. The intervention started with a training phase supported by the physiotherapist. Intervention part A consisted of three physiotherapeutic sessions of 30 min duration, at intervals of four weeks and daily self-supported exercises by the subjects at home and continued until T1 (3 months after start of the intervention). At T1 a 3-months training phase without the support of the physiotherapist began, with subjects’ self-supported, daily physiotherapeutic training at home until T2 (intervention part B). After T2, subjects were instructed to stop the exercises until final evaluation at T3, 6 months after T2 (Supplementary Material I) [78].

The MaMuT program consisted of three physiotherapeutic exercises. (Supplementary Material II, [78]) Exercise 1 aimed to increase bite force and chewing efficiency by improving coordination. Subjects were asked to chew a cube with 1 × 1 cm edge length (material: 3M™ Permadyne Penta H, Espe) daily for five training units of 30 s each interrupted by a 2-min break. The isometric exercises 2 and 3 aimed to build up strength in the masticatory muscles [78].

All subjects received written and illustrated instructions with the exercises as well as the necessary material: an hourglass for monitoring the duration of the training per exercise and the duration of the break, as well as chewing cubes to train the bite force. In each physiotherapeutic session, the physiotherapist monitored whether the subjects were able to perform the exercises independently and correctly. Adherence to study participation was assessed by these means. All subjects were able to perform all exercises independently at each appointment with the physiotherapist. Further verification of adherence to the intervention was not wanted because the study was also intended to be oriented toward everyday life. There is always the possibility with home therapeutic instructions that these are carried out by some subjects with much or little vigor, or sometimes they are not practiced at home at all. In the study, no ideal situation should be pursued that would later have no relevance in real-life application. The subjects or their relatives/caregivers were motivated, re-instructed and corrected if necessary.

### 2.5. Statistical Considerations

Because no literature on endpoints was available, power calculations were not performed in this pilot study. The sample size was determined based on similarly designed studies and the sample sizes used in them [84,85].

The statistical analysis corresponded to the pilot character of the study. Where appropriate, graphical tools, and descriptive statistics were used. To account for the temporal dependency structure and to control for age and gender, mixed linear models were tentatively used. If parametric model assumptions were violated, robust non-parametric tests were used for the comparison between mental state and intervention effect. Intention to Treat (ITT) and Per Protocol (PP) analyses were performed to account for potential deviations of the research protocol. Dropouts were replaced with new recruitments [77].

Subjects who died during the study had to be excluded from the analysis. For the longitudinal analysis only, subjects who attended at least three out of four evaluation time points were taken into consideration. All statistical analyses and plots were computed with the statistical software R [86], including the packages tidyverse [87], mice [88] and missForest [89] or SPSS 23.0 for descriptive statistics [90]. Missing values were identified and imputed using the missForest algorithm [91].

To describe the outcome of the intervention in the experimental groups, the differences between time points for VOH and MOF were calculated as ∆VOH and ∆MOF. In this regard, the difference between T1 and T0 refers to the intervention part A—MaMuT program with physiotherapeutic training of the subjects under the direct guidance of a physiotherapist (once every four weeks) in combination with daily, self-supported exercises by the subjects at home, the difference between T2 and T0 refers to the intervention part B—MaMuT program followed by subjects’ self-supported, daily physiotherapeutic training at home without further physiotherapeutic support. For the control groups, the differences ∆VOH and ∆MOF were calculated as T3 minus T0 and serve as measures of the changes over time without participation in an intervention. Positive ∆VOH indicate reduced chewing efficiency and vice versa. Positive ∆MOF indicate an increase in chewing efficiency and vice versa.

### 2.6. Ethical Consideration

The study met the standards of the Declaration of Helsinki and of good clinical practice and was approved by the competent Cantonal Ethics Committee (CEC) of Zurich (KEK-ZH 2017-00363). All subjects or their legal representatives gave written informed consent.

## 3. Results

### 3.1. Study Population

A total of 71 subjects (male 33.8%, mean age 78.3 ± 9.3 years) were included. One participant was excluded due to death before the study was completed (drop-out *n* = 1; 1.4%). Results of socio-demographic, geriatric and dental items are shown in the Supplementary Material III.

There are minor differences in chewing efficiency (VOH) between the ConG and the ExpG at T0. The differences at T0 are greater for the maximum occlusal force (MOF) between the ConG and the ExpG (Table 1).

### 3.2. Changes in Chewing Efficiency (VOH), Subjective Chewing Efficiency Assessment Scale (SAS), and Maximum Occlusal Force (MOF) over Time

**Figure 1:**
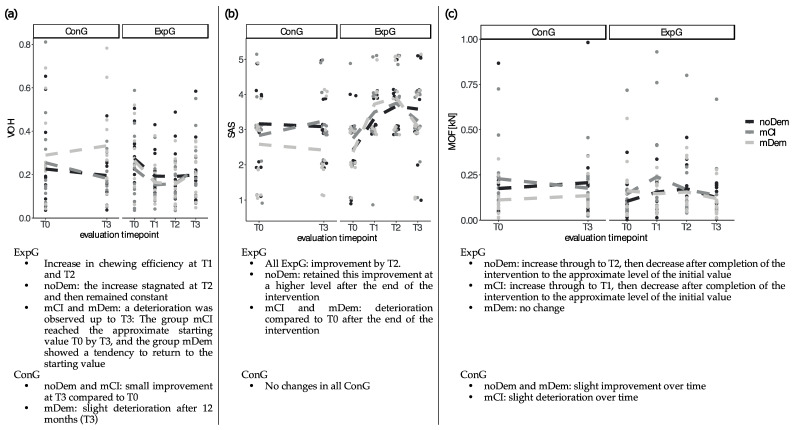
Visualization of changes in (**a**) chewing efficiency (Variance of Hue, VOH), (**b**) chewing efficiency subjective assessment scale (SAS) and (**c**) maximum occlusal force (MOF in kN) over time (evaluation timepoints T0–T3 (T0—baseline, T1—3 months and T2—6 months after starting the intervention (experimental group only), T3—12 months after T0)); (noDem—no dementia, mCI—mild cognitive impairment, mDem—mild dementia, ConG—control group, ExpG—experimental group). The different colored dots correspond to the individual measurements per subgroup (noDem, mCI and mDem). The short lines illustrate the changes of the individual subgroups in the mean over time.

The changes in VOH, SAS, and MOF over time (evaluation timepoints T0–T3) are presented in Table 1 and Figure 1. Overall, an increase in VOH and SAS over time was observed for all ExpG during the intervention. In the ExpG noDem and mCI an increase in the MOF was observed during intervention followed by a decrease after completion of the intervention to the approximate level of the initial value. The ExpG mDem showed no change in MOF over time (Table 1, Figure 1).

### 3.3. Differences in Chewing Efficiency (VOH), and Maximum Occlusal Force (MOF) between the Evaluation Time Points

**Figure 2:**
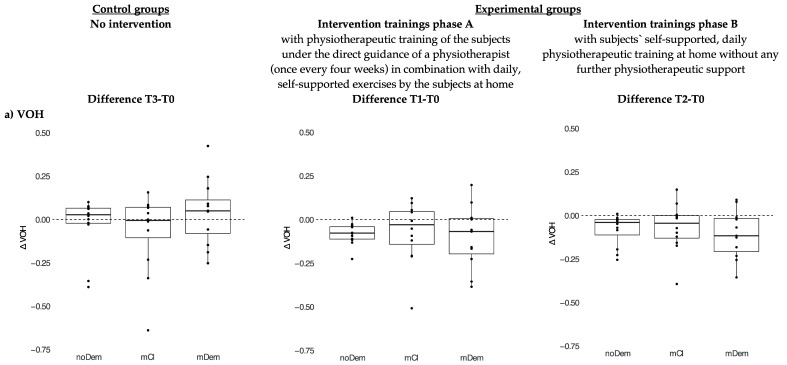

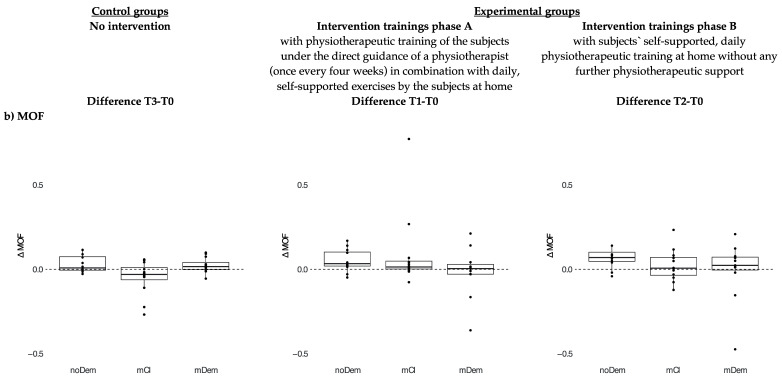
Visualization of the differences in (**a**) chewing efficiency (Variance of Hue, VOH) and (**b**) maximum occlusal force (MOF) as an expression of the changes over time in subjects without the intervention of the MaMuT (masculatory muscle training) program (control groups) and in subjects with the MaMuT program (experimental groups) (subgroups: noDem—no dementia, mCI—mild cognitive impairment, mDem—mild dementia). The differences between time points for VOH and MOF are calculated as ∆VOH and ∆MOF. VOH—negative differences as an expression of improvement of the chewing efficiency and vice versa/MOF—positive differences as an expression of improvement of the bite force and vice versa.

The differences in VOH and MOF, i.e., the differences in the VOH and MOF for ConG between T3 and T0 and for ExpG between T1-T0 (intervention part A) and T2-T0 (intervention part B), as an expression of the changes over time in subjects without the intervention of the MaMuT (masticattory muscle training) program (control groups) and in subjects with the MaMuT program (experimental groups), were calculated. (Figure 2) The differences between time points for VOH and MOF were calculated as ∆VOH and ∆MOF.

In all ExpG, the median difference in VOH for both interventions part A and part B was negative, which corresponds to an improvement in chewing efficiency over time. The median of ExpG mDem was lower indicating a greater improvement in chewing efficiency between T2 and T0 (Figure 2a) than in ExpG noDem and mCI. The ∆VOH values in all ConG (T3–T0) confirmed a deterioration in chewing efficiency (ConG noDem and mDem) and a similar chewing efficiency in ConG mCI (Figure 2a).

The ∆MOF values (T1–T0 and T2–T0) in the ExpG noDem and mDem indicated an improvement in MOF over the entire intervention period (difference T2–T0). For the ConG noDem and mDem no change resp. a slight deterioration of the MOF in ConG mCI was observed (Figure 2b).

## 4. Discussion

In this study, the chewing efficiency of all subjects was improved, while people with mild dementia appeared to benefit most from the physiotherapeutic exercises. Bite force increased only in subjects with no cognitive impairment or mild cognitive impairment. It was shown that physiotherapeutic exercises were effective in people with onset of dementia and mild dementia as well as in people without cognitive impairment. Therefore, the authors must reject their two initial hypotheses.

### 4.1. Study Limitation

Subjects with more severe dementia (MMS ≤ 17) had to be excluded from the intervention due to the expected lack of ability to follow the instructions and carry out the exercises.

The simple and time-efficient Mini Mental State Examination (MMSE) is considered a suitable instrument to quantify cognitive deficits and assess the severity of cognitive impairment [91]. However, there may be a bias, since subjects with cognitive impairments may already know the questions of the MMSE from previous occasions which may have an influence on results. Furthermore, using the MMSE it is not possible to differentiate between types of dementia. The sensitivity, especially in mild dementia, is questionable.

All examinations of the study were conducted by a non-blinded investigator which may have caused a bias. Nevertheless, the authors assume that the advantages of having only a single investigator, for the study outweigh the disadvantages by improving the consistency of the investigations. Subjects with cognitive impairments are more responsive to a well-known, familiar person and participate more effectively in the survey. In addition, within the setting of a long-term care facility, it is also advantageous to have only one contact person for the nursing staff involved to clarify inquiries in a targeted manner.

Physiotherapeutic treatment was provided by a professional physiotherapist throughout this study. It would have been desirable to have the possibility to control the individual, home-based training of the subjects on a daily or at least weekly basis, or to have this training professionally supervised. Due to the number of subjects and the personnel, time and financial resources involved, this was not possible. The majority of the subjects were very old, frail and in need of care. This results in limitations regarding the feasibility of the evaluation of bite force and chewing efficiency.

In the present study, it was possible to ensure that caregivers in long-term care facilities carried out the daily exercises together with the subjects. For this purpose, the nurses were instructed by the physiotherapist and were present at every physiotherapeutic session if necessary. In situations where cooperation with the participating long-term care facility is not as easy, it may be doubtful whether training on a daily basis would be possible, e.g., due to a lack of time of the caregivers or willingness of the subjects. In subjects who live independently at home, it was not possible to control compliance. Therefore patients were asked to independently demonstrate the MaMuT exercises during physiotherapy sessions to assess their performance. It turned out that subjects were generally able to perform the exercises from memory and on their own. These subjects mostly belonged to the noDem or mCI group. If minor corrections in the execution were necessary, these were discussed with the subjects by the physiotherapists. In countries, where funding is available and therapists offer home visits, MaMuT could be offered at patients’ homes.

The slight improvement in bite force and chewing efficiency in the control groups may be related to learning effect [92] and subjects becoming used to the equipment employed despite a measurement interval of 12 months [37]. Various studies have reported this effect when measurements were carried out t short intervals [37,38,68].

The chewing ability decreases with age (subjects with mild dementia were the oldest subjects) [42,93,94]. In addition, problems in people aged 70 years and older, such as a decline in physical performance and a deterioration in oral function, might be decisive [95,96]. Additionally, the subjects with mild dementia had the highest degree of need for care compared to the other subjects, thus making functional limitations more likely. There is a probability that the MaMuT program was as effective in the mild dementia group but the measurement method used (Occlusal Force Meter GM10) was not suitable for effectively displaying slightest improvements, especially in bite force. The literature shows that e.g., psychological factors, such as fear of dental damage, can have an influence on the measurement [19,97,98]. Other authors discuss other factors, such as location of the bite force recording within the dental arch, as well as the extent of the vertical separation of the teeth and the jaws due to the bite fork. Additionally, the state of dentition, the mental state during the evaluation, and the investigator’s and subjects’ attitude, as well as malocclusions and signs and symptoms of temporomandibular dysfunction are discussed as influencing factors [98,99,100,101,102]. It might be possible that the measurement method itself was less effective in subjects with mild dementia 12 months after the start of the study. Often the physiological and mental state of people in need of care changes very rapidly. Such changes could have had a negative effect on the measurement. A reduced adherence to therapy or to the evaluation should also be discussed. Subjects with mild dementia, may only be able to conduct daily individual exercises with the support of their caregivers. It is possible that these subjects showed less compliance than subjects of other groups due to a limited understanding of the exercises and their purpose. Compliance of subjects with dementia may change from day to day. However, the evaluation of the measured values was only carried out on a single day regardless of the subjects’ condition and was not repeated on another day if cooperation was limited.

Another limitation of the study is that factors such as the number of remaining teeth in the posterior region, the loss of vertical dimension [103] or the presence and type of denture present (fixed vs. removable) [104] were not considered in the analysis. This could result in a change in the masticatory function outcome as well as on influences on the afferent trigeminal signals toward the central nervous system (CNS). However, the aim of the present study was to develop a training program suitable for everyday use and to measure its effectiveness as a function of cognitive function without considering other influencing factors. This should be considered in further studies.

### 4.2. Comparison with Other Studies

A direct comparison of the data of the present study with other studies is not possible, as this is, to the best knowledge of the authors, the first study to have carried out a masticatory muscle training with a combination of isometric and isotonic exercises in subjects with and without dementia. Most of the studies investigating bite force improvements through training have examined young, healthy subjects (e.g., [37,38,71]). The influence of increasing physical frailty and reduced cognitive abilities on the outcome in this study is likely.

Thompson et al. concluded that bite force may be increased easily by means of isometric training. However, they assume that an actual strengthening of the masticatory muscles is more difficult to achieve [38]. Kiliaridis et al. also showed that four-week isotonic training with a hard chewing gum could increase both the functional capacity and the strength of the masticatory muscles. An increase in fatigue resistance due to the training was not observed [37]. The assumption has been made that training with hard chewing gums, as used in the present study, is particularly effective in increasing bite force and saliva flow [72]. However, only a few studies have observed the effect of gum hardness on masticatory performance [69,70,73].

In the present study, both isometric and isotonic exercises were used to increase bite force and improve chewing efficiency. Isometric exercise is known to be effective in increasing the strength of limb muscles. Isometric exercises may, therefore, also be suitable for strengthening the jaw muscles [105,106,107,108,109]. It is generally accepted that an isometrically trained muscle measured during isometric contractions will show the greatest training effect [110]. Nevertheless, He et al. assume that training that includes both isometric and isotonic exercises might be more effective in increasing the masticatory muscle strength [71]. In this study, the period of the training phase was designed to be longer than reported in other publications (e.g., [72]). A long-term observation of more than six months, as carried out in this study, should be aimed for in follow-up studies, in order to be able to observe long-term effects after the end of the training phase and, if necessary, to be able to exclude confounders such as increasing frailty or dementia.

The maximum occlusal force (MOF) was included in the present analysis, due to the observation made by the investigator during the evaluation that the initial measurement was often lower than the one that followed (a possible cause is test subjects becoming used to the device/method). The last measurement was often lower than the previous one (possible fatigue effect). In the present study, no increase in bite force was observed in subjects with mild dementia through participation in the MaMuT program. A possible cause for this may be that the subjects were unable to train effectively with the hard and less elastic chewing material used. A study by Nakagawa et al. suggested to start training of the masticatory muscle using a soft gum and then later switch to a harder gum when the subjects are already used to gum chewing [72].

Sarcopenia is a widespread and serious condition in geriatric patients. The reduction in muscle mass does not exclude the chewing muscles, so these should also be considered when researching malnutrition. That a muscle training can have a positive effect is also known from other muscle groups. Thus, to maintain the ability to walk, geriatricians demand daily training outside the home of approximately 30 min [111] or additional 1000 steps per day [112]. Similarly, the results of the current study support a recommendation for daily training of the chewing muscles of seniors with and without cognitive impairment or dementia. Thus, seniors should be encouraged to chew gum intensively for 15 min twice a day—after breakfast and after lunch. For a widespread implementation, it would also be necessary to clarify to what extent the costs of professional physiotherapeutic exercises are covered by health insurance. Similar examinations could be carried out in an inpatient setting, where daily individual physiotherapeutic work with the participant can be guaranteed.

In addition to strengthening the chewing muscles and improving chewing efficiency, another positive effect would be an increase in saliva production and thus improved remineralization of the enamel after the meal which usually involves an acid attack by various foods. However, a problem with this type of training is that the oldest old in particular are often averse to gum chewing. Furthermore, chewing gum may be more difficult for denture wearers because it sticks to the dentures. It is also important to be aware that people with dementia should chew under supervision to reduce the risk of swallowing or aspirating the chewing gum.

### 4.3. Measurement Methods

The bite force is greatest at a vertical distance of up to 15–20 mm jaw opening [113]. The jaw opening in the present study was approximately 5 mm due to the measuring device (Occlusal Force Meter GM10™). The objective measurement of the chewing efficiency with the two-color mixing test according to Schimmel provides a simple, and reliable method [114], which in the present study was also applied in the setting of a long-term care facility.

When using SAS to determine chewing efficiency, it is unclear whether human eye can judge the degree of color graduation as precisely as a machine. Alternatively, it is possible to evaluate the color mixture using a smartphone camera. However, higher degrees of color mixing result in a lower precision [115].

Based on the present study, the authors pose the following questions, which need to be clarified in future studies. The exact reasons why people with mild dementia could not achieve an increase in bite force despite an improvement in chewing efficiency must be identified. Furthermore, more attention should be paid to improving masticatory performance through physiotherapeutic exercises in older people and in people with dementia in general. Chewing can be considered to be the basis of food intake. A healthy diet and the ability to chew food without limitations contribute to improve the quality of life and the nutritional status and therefore the general health. It must be clarified whether targeted, physiotherapeutically guided, regular training of the masticatory muscles has an effect on direct and indirect nutritional parameters (e.g., albumin level, body mass index, walking speed).

An essential factor for clarifying the effectiveness of physiotherapeutic training of the masticatory muscles is the question to what extent the applied evaluation methods can be used to measure bite force and chewing efficiency in people with dementia (including moderate and severe dementia) and whether alternatives should be researched. Certainly, it would also be helpful to clarify to what extent physiotherapists are trained in everyday life to train masticatory muscle building with seniors.

## 5. Conclusions

There is a difference in training effect of the masticatory muscles depending on the level of cognitive function. Without further training, however, the effect disappears.

The MaMuT program with physiotherapeutic support is a potential method for improving masticatory performance in people with cognitive impairment and mild dementia. However, the effectiveness of the method described should be critically considered in terms of limitations of the study, the study population, and the duration of the observation period. Thus, the results of this study should serve as a starting point for further investigations of factors that influence masticatory performance, the suitability of the measuring instruments and for the development of therapeutic approaches for improved nutritional status in people with dementia.

## Figures and Tables

**Table 1:** Maximum occlusal force (MOF, in N) und chewing efficiency (Variance of Hue, VOH) separated by evaluation time-point (T0–T3) and evaluation group (group 1–3) respectively subgroup. (noDem—no dementia, mCI—mild cognitive impairment, mDem—mild dementia, ConG—control group, ExpG—experimental group, SD—standard deviation, IQR—interquartile range.

			Maximum Occlusal Force [N]				Chewing Efficiency as Variance of Hue [VOH]			
			T0	T1	T2	T3	T0	T1	T2	T3
Group 1 noDem MMSE 30-28	ConG [*n* = 12]	Mean/±SD	175 ± 229			207 ± 265	0.225 ± 0.211			0.195 ± 0.120
		Median	85			102	0.169			0.198
		IQR	139			161	0.204			0.140
	ExpG [*n* = 12]	Mean/±SD	105 ± 103	155 ± 125	173 ± 130	125 ± 59	0.273 ± 0.126	0.193 ± 0.104	0.192 ± 0.134	0.199 ± 0.148
		Median	82	132	145	105	0.254	0.157	0.140	0.165
		IQR	78	150	140	98	0.150	0.109	0.141	0.134
Group 2 mCI MMSE 27-25	ConG [*n* = 12]	Mean/±SD	229 ± 189			177 ± 112	0.255 ± 0.216			0.182 ± 0.103
		Median	164			141	0.161			0.167
		IQR	113			130	0.197			0.125
	ExpG [*n* = 12]	Mean/±SD	146 ± 186	239 ± 302	164 ± 213	141 ± 182	0.226 ± 0.170	0.153 ± 0.089	0.172 ± 0.089	0.228 ± 0.143
		Median	82	96	85	87	0.194	0.135	0.194	0.192
		IQR	85	162	78	71	0.197	0.091	0.112	0.147
Group 3 mDem MMSE 24-18	ConG [*n* = 12]	Mean/±SD	118 ± 103			163 ± 84	0.289 ± 0.194			0.312 ± 0.212
		Median	76			154	0.235			0.256
		IQR	134			57	0.079			0.149
	ExpG [*n* = 11]	Mean/±SD	163 ± 163	154 ± 90	156 ± 138	120 ± 102	0.264 ± 0.144	0.164 ± 0.091	0.156 ± 0.107	0.216 ± 0.114
		Median	78	159	94	78	0.241	0.153	0.135	0.256
		IQR	159	152	178	140	0.174	0.104	0.175	0.196

## Data Availability

The data presented in this study are available on request from the corresponding author. The data are not publicly available due to ethical reasons.

## Supplementary Materials

The following supporting information can be downloaded at: https://www.mdpi.com/article/10.3390/ijerph19073778/s1, Supplementary Material I: Timetable for evaluation points and intervention time points; Supplementary Material II: MaMuT program. Instructions for study participants; Supplementary Material III: Overview on socio-demographic items, geriatric and dental assessments of all subjects.
