# Supplementary material for: The Effect of a Masticatory Muscle Training Program on Chewing Efficiency and Bite Force in People with Dementia
Source: Int J Environ Res Public Health. 2022 Mar 22;19(7):3778. doi: 10.3390/ijerph19073778 (PMC8997984; doi:10.3390/ijerph19073778)
Supplement: Supplementary file 1 [file ijerph-19-03778-s001.zip › Supplementary material I.pdf]

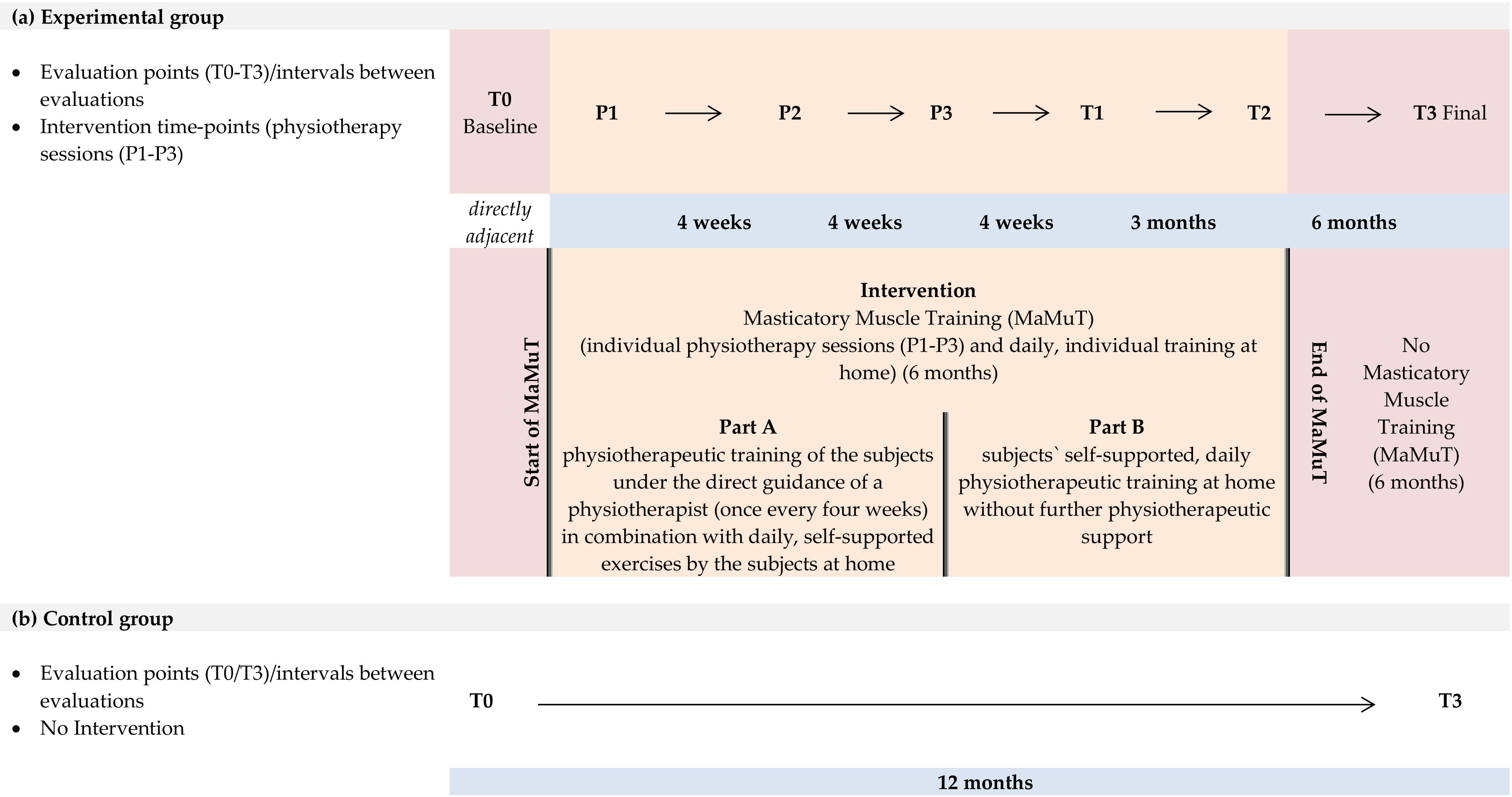

Figure S1. Timetable for evaluation points (T0-T3) and intervention time-points (P1-P3, if applicable) for a) experimental group and b) control group. MaMuT – masticatory muscle training
