# Supplementary material for: The Effect of a Masticatory Muscle Training Program on Chewing Efficiency and Bite Force in People with Dementia
Source: Int J Environ Res Public Health. 2022 Mar 22;19(7):3778. doi: 10.3390/ijerph19073778 (PMC8997984; doi:10.3390/ijerph19073778)
Supplement: Supplementary file 1 [file ijerph-19-03778-s001.zip › Supplementary material III.pdf]

Table S1. Overview on socio-demographic items, geriatric and dental assessments of all subjects. (noDem – no dementia, mCI – mild cognitive impairment, mDem – mild dementia, ConG control group, ExpG – experimental group).

		All Subjects [n=71]	Group 1 - noDem MMSE 30-28		Group 2 - mCI MMSE 27-25		Group 3 - mDem MMSE 24-18	
			ConG [n=12]	ExpG [n=12]	ConG [n=12]	ExpG [n=12]	ConG [n=12]	ExpG [n=11]
**Socio-demographic items**								
**Sex [n/%]**	Male	24 / 33.8	6 / 50.0	5 / 41.7	4 / 33.3	4 / 33.3	1 / 8.3	4 / 36.4
**Age [years]**	Mean (±SD)	78.3 (±9.3)	75.2 (±9.7)	73.1 (±6.9)	78.7 (±12.2)	78.9 (±8.6)	82.9 (±6.1)	81.4 (±9.0)
	Median (Range)	79 (61-95)	76 (62-92)	74.5 (63-83)	83 (61-95)	78 (65-94)	83.5 (71-93)	85 (65-95)
**In need of care [n / %]**		30 / 42.3	0 / 0	1 / 8.3	5 / 41.6	5 / 41.6	10 / 83.3	9 / 81.8
**Geriatric Assessment**								
**Mini Mental State Examination (MMSE)**	Median (Range)	27 (18-30)	29 (28-30)	29 (28-30)	27 (25-27)	26.5 (25-27)	21.5 (18-24)	21 (18-24)
**Barthel-Index (ADL)**	Median (Range)	100 (25-100)	100	100	100 (50-100)	100 (30-100)	100 (25-100)	90 (55-100)
**Mini nutritional assessment (MNA)**	Median (Range)	24.5 (14.5-30)	26.75 (19-27.5)	25.75 (15.5-27.5)	24.75 (17-28)	24.75 (19.5-30)	21.75 (14.5-24.5)	23.5 (19-25.5)
**Body Mass Index (BMI) [kg/m2]**	Mean (±SD)	25.9 (±4.8)	26.6 (±3.9)	28 (±4.8)	24 (±3.8)	26.6 (±4.5)	23.6 (±4.8)	26.6 (±6.1)
	Median (Range)	25.4 (17.6-38.3)	26.3 (21-32.7)	27.9 (21.7-38.3)	23.8 (18.7-29.4)	25.2 (22.4-37.1)	22.5 (17.6-32.4)	24.7 (17.9-37.1)
**Dental Assessment**								
**Number of teeth related to 28 teeth [n]**	Mean (±SD)	16.5 (±7.3)	15.8 (±6.4)	11.9 (±7.7)	18.8 (±6.4)	15 (±8.0)	18.7 (±7.3)	18.7 (±6.9)
	Median (Range)	16 (2-28)	14 (8-28)	10.5 (3-23)	19.5 (4-26)	16 (2-28)	22 (5-28)	19 (9-28)
**Number of support zones** Median (Range)		4 (1-4)	4 (2-4)	4 (3-4)	4 (2-4)	4 (1-4)	4 (1-4)	4 (2-4)
**Tooth and denture status <u>upper jaw</u> [n / %]**								
	Fully dentate, no dentures or fixed dentures	6 / 8.5	1 / 8.3	1 / 8.3	1 / 8.3	1 / 8.3	1 / 8.3	1 / 9.1
	Partially dentate, fixed dentures	18 / 25.4	1 / 8.3	1 / 8.3	4 / 33.3	2 / 16.7	6 / 50.0	4 / 36.4
	Partially dentate, removable denture	28 / 39.4	8 / 66.7	5 / 41.7	3 / 25.0	6 / 50.0	4 / 33.3	2 / 18.2
	Partially dentate, no dentures available	11 / 15.4	1 / 8.3	1 / 8.3	3 / 25.0	2 / 16.7	1 / 8.3	3 / 27.3
	Edentulous, removable dentures	8 / 11.3	1 / 8.3	4 / 33.3	1 / 8.3	1 / 8.3	0 / 0	1 / 9.1
	Edentulous, no dentures available	0 / 0	0 / 0	0 / 0	0 / 0	0 / 0	0 / 0	0 / 0
**Tooth and denture status <u>lower jaw</u> [n / %]**								
	Fully dentate, no dentures or fixed dentures	6 / 8.5	1 / 8.3	0 / 0	0 / 0	1 / 8.3	2 / 16.7	2 / 18.2
	Partially dentate, fixed dentures	19 / 26.7	4 / 33.3	0 / 0	5 / 41.7	1 / 8.3	6 / 50.0	3 / 27.3
	Partially dentate, removable denture	31 / 43.7	6 / 50.0	9 / 75	2 / 16.7	6 / 50.0	4 / 33.3	4 / 36.4
	Partially dentate, no dentures available	15 / 21.1	1 / 8.3	3 / 25	5 / 41.7	4 / 33.3	0 / 0	2 / 18.2
	Edentulous, removable dentures	0 / 0	0 / 0	0 / 0	0 / 0	0 / 0	0 / 0	0 / 0
	Edentulous, no dentures available	0 / 0	0 / 0	0 / 0	0 / 0	0 / 0	0 / 0	0 / 0
